# Effect of vacancy concentration on the lattice thermal conductivity of CH_3_NH_3_PbI_3_: a molecular dynamics study†

**DOI:** 10.1039/d1ra05393k

**Published:** 2021-10-20

**Authors:** Song-Nam Hong, Chol-Jun Yu, Un-Gi Jong, Song-Hyok Choe, Yun-Hyok Kye

**Affiliations:** Computational Materials Design, Faculty of Materials Science, Kim Il Sung University Ryongnam-Dong, Taesong District Pyongyang Democratic People’s Republic of Korea cj.yu@ryongnamsan.edu.kp

## Abstract

Hybrid halide perovskites are drawing great interest for photovoltaic and thermoelectric applications, but the relationship of thermal conductivities with vacancy defects remains unresolved. Here, we present a systematic investigation of the thermal conductivity of perfect and defective CH_3_NH_3_PbI_3_, performed using classical molecular dynamics with an *ab initio*-derived force field. We calculate the lattice thermal conductivity of perfect CH_3_NH_3_PbI_3_ as the temperature increases from 300 K to 420 K, confirming a good agreement with the previous theoretical and experimental data. Our calculations reveal that the thermal conductivities of defective systems at 330 K, containing vacancy defects such as V_MA_, V_Pb_ and V_I_, decrease overall with some slight rises, as the vacancy concentration increases from 0 to 1%. We show that such vacancies act as phonon scattering centers, thereby reducing the thermal conductivity. Moreover, we determine the elastic moduli and sound velocities of the defective systems, revealing that their slower sound speed is responsible for the lower thermal conductivity. These results could be useful for developing hybrid halide perovskite-based solar cells and thermoelectric devices with high performance.

## Abstract 2

Molecular dynamics simulations with the MYP force field were performed to determine the thermal conductivity of perfect and defective bulk MAPbI_3_. Thermal conductivity was found to decrease overall as the vacancy concentration increased.

## Graphical abstract

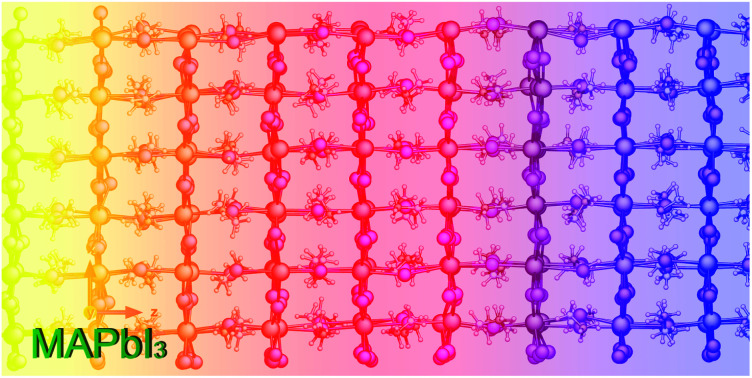

## 1 Introduction

Halide perovskites (HPs) have emerged as exciting new materials for photovoltaic applications owing to their unique properties of strong light absorption, high carrier mobility and relatively simple manufacturing process.^1^ In fact, the power conversion efficiency of perovskite solar cells (PSCs), which use HPs as a light absorber, has rapidly increased from an initial value of 3.8% in 2009 (ref. 1) to a certified value of 25.5% in 2020.^2,3^ In addition to photovoltaics, HPs draw great interest in applications such as thermoelectrics,^4–6^ light emitting diodes,^7^ photodetectors^8^ and lasers.^9^ For these applications, the lattice thermal conductivity of the HPs is the most important quantity to consider for obtaining the best performance under the conditions these devices are normally operated.^10^ Moreover, as the temperature of devices using HPs could increase rapidly during operation, HPs can be decomposed into their constituent components, leading to degradation of device performance. Meanwhile, a temperature difference between the sandwiched layers of HP-related devices can cause mechanical stress, resulting in device destruction. Therefore, it is necessary to gain insight into the thermal conductivity of HPs for guaranteeing the normal operation of device and designing thermoelectric materials.

The chemical formula of HPs is written as ABX_3_, where A is the monovalent organic or inorganic cation, such as CH_3_NH_3_^+^ (or MA^+^), CH(NH_2_)_2_^+^ (or FA^+^) or Cs^+^; B is the divalent metallic cation, such as Pb^2+^, Sn^2+^ or Ge^2+^, and X is the halogen anion such as I^−^, Br^−^ or Cl^−^. Among them, a prototype HP material used in most applications is methylammonium lead iodide CH_3_NH_3_PbI_3_ (or MAPbI_3_). In experiments, MAPbI_3_ was found to have an ultralow thermal conductivity *κ* of 0.3–0.5 W m^−1^ K^−1^ at room temperature,^5,6,11–14^ which is useful for thermoelectric and thermal barrier applications. In line with experiments, theoretical calculations with density functional theory (DFT)^15–17^ and/or classical molecular dynamics (MD) methods^17–20^ have also been performed, revealing that the *κ* value for MAPbI_3_ is in the range 0.2 to 1.8 W m^−1^ K^−1^, depending on the crystalline phase and calculation method. It should be noted that the calculations mainly focused on uncovering the mechanism of its ultralow *κ*, demonstrating that the MA^+^ cation plays a critical role in suppressing *κ*, as confirmed in infrared and Raman spectroscopy experiments.^21–23^

In general, HPs are synthesized from precursor solutions under ambient conditions by simple and low-cost chemical processing, so that various defects are inevitably created in the resultant HP solid.^24,25^ Such defects, including vacancies, antisites and interstitials, were found to affect the optoelectronic and thermal properties of HPs. Therefore, numerous investigations have been performed on the unusual defect physics and chemistry of HPs, demonstrating that they are defect-tolerant even though the defect concentration is as high as 10^17^–10^20^ cm^−3^ due to their low formation energies.^26–28^ It was known that defects usually create deep levels near the mid gap, acting as charge carrier traps and accordingly deteriorating the performance in the Si- or iii–v semiconductor-based solar cells. In PSCs, however, defects fortunately act as shallow donors or acceptors,^29,30^ thus not severely degrading the optoelectronic properties and charge carrier mobilities of HPs.^31,32^ In spite of many investigations on the electronic transport properties of MAPbI_3_, the effect of vacancy defects on its lattice thermal and mechanical properties remains unexplored.

In this work, we investigate the effects of various vacancy defects with different concentrations on the thermal conductivity of MAPbI_3_ by performing systematic MD simulations. From the simulation data, we determine the contributions of individual MA^+^, Pb^2+^ and I^−^ ions to the change of heat transport properties. We estimate the elastic constants and sound speeds in perfect and defective systems, revealing their relation with the thermal conductivity. Section 2 describes the MD method used to calculate the thermal conductivity, simulation models with diverse vacancies, and the employed force field. Section 3 presents the simulation results and discussion, comparing them with the available experimental and theoretical data. Finally, conclusions are provided in Section 4.

## 2 Computational methods

### 2.1 Molecular dynamics methods

Thermal conductivity of a crystalline solid is expressed as a second-order tensor, which can be calculated using different kinds of MD methods, such as equilibrium MD (EMD)^33^ and approach-to-equilibrium MD (AEMD).^34^ The EMD approach is based on the fluctuation–dissipation theory at an equilibrium state of system, where the thermal conductivity can be calculated by integrating the heat flux autocorrelation function (HFACF) obtained with a sufficiently long-term run and using the following Green–Kubo relation,^33^1
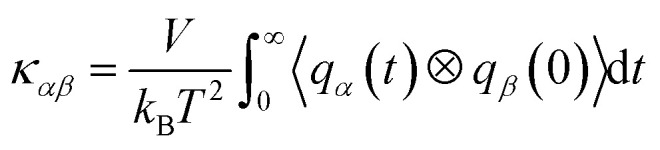
where *k*_B_ is the Boltzmann constant, *V* is the volume of system, and *α* and *β* are the Cartesian coordinates. The second-order tensor *ϕ*_*αβ*_(*t*) = 〈*q*_*α*_(*t*) ⊗ *q*_*β*_(0)〉/〈*q*_*α*_(0) ⊗ *q*_*β*_(0)〉 is the HFACF, and *q*_*α*_(*t*) is the heat flux written as,2
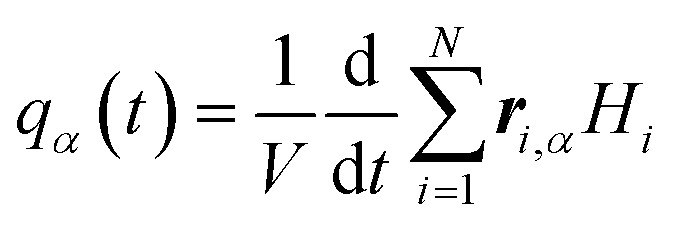
where ***r***_*i*_ is the position vector of the *i*th atom, and *H*_*i*_ is the Hamilton function of the system.

**Fig. 1:**
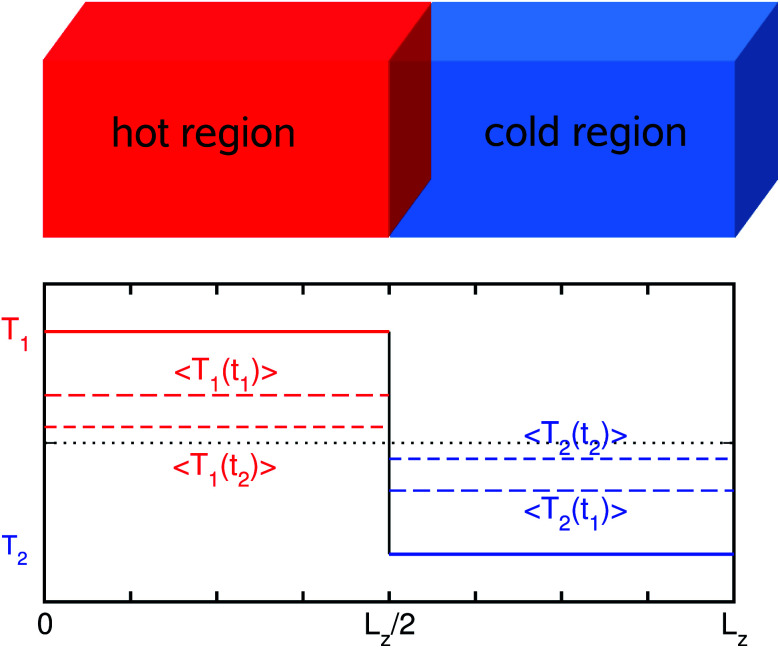
Temperature profile during the AEMD simulation. After enough equilibration at temperature (*T*_1_ + *T*_2_)/2, the simulation box is split into two regions, *i.e.*, hot and cold regions, which are equilibrated at *T*_1_ and *T*_2_. Here, *T*_1_ > *T*_2_, and 〈*T*_1_(*t*_1_)〉 and 〈*T*_2_(*t*_2_)〉 are the average temperatures of the regions with *t*_1_ < *t*_2_. *L*_*z*_ is the length of the simulation box in the *z*-direction.

The AEMD method yields *κ* by solving the heat flux equation along the flux direction *z*,^34^3
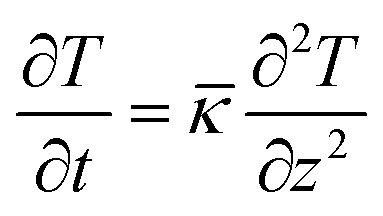
where *κ̄* = *κ*/*ρC*_V_ is the thermal diffusivity with mass density *ρ* and volumetric heat capacity *C*_V_. For the first step, the two sub-regions are equilibrated with a periodic boundary condition at different temperatures as depicted in Fig. 1. Next, an NVE simulation is performed for the entire system, gradually decreasing the difference of average temperatures between the two sub-regions. Then, the temperature difference is fitted to the exponential function, giving the thermal diffusivity *κ̄* (see the ESI for details†).

### 2.2 Modeling and computational details

The MAPbI_3_ crystal was found to be stabilized in the orthorhombic phase below 160 K, the tetragonal phase between 160 and 330 K, and the cubic phase over 330 K.^35,36^ Since the MAPbI_3_ crystal is heated for calculating the *κ* value of MAPbI_3_ above room temperature, we adopted the tetragonal phase with *I*4/*mcm* space group at temperatures below 330 K and a pseudocubic phase with *Pm*3̄*m* space group at higher temperatures.

**Fig. 2:**
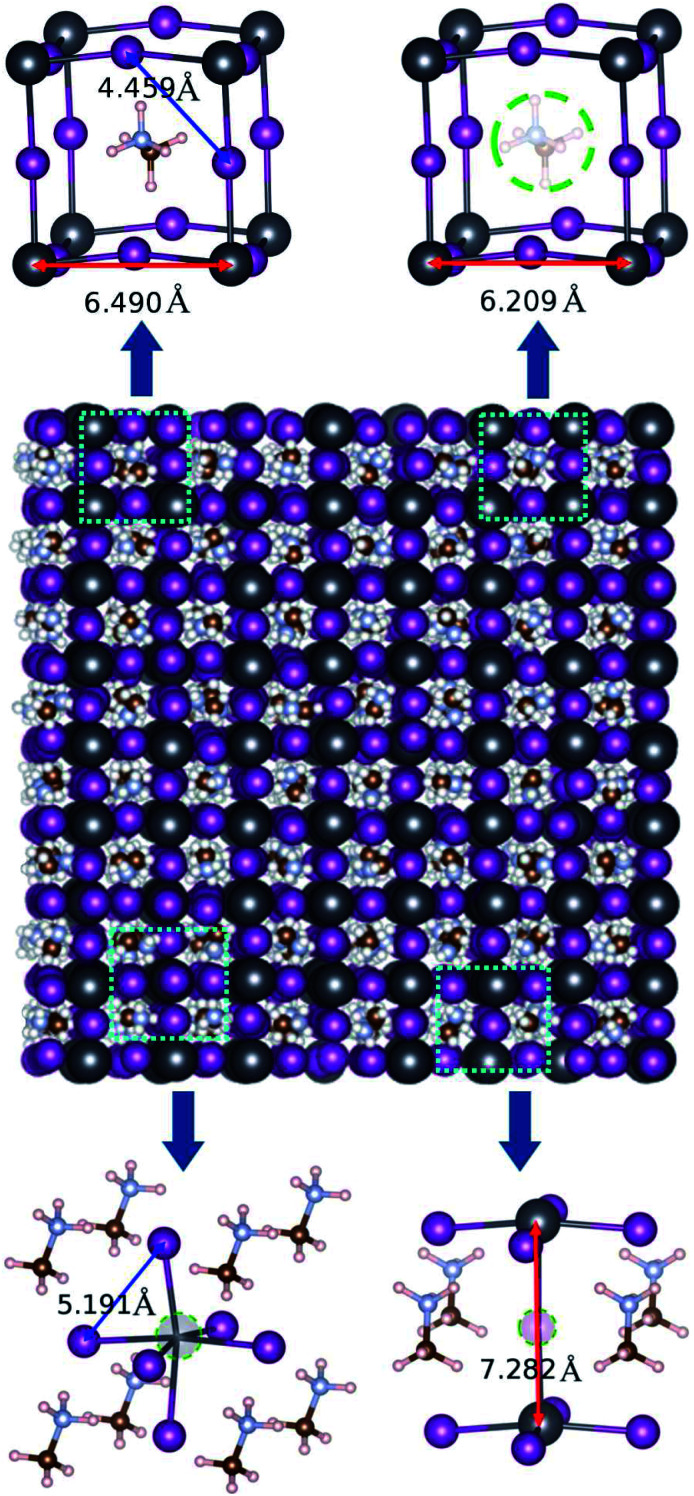
Space-filling view of the 8 × 8 × 8 supercell for a pseudocubic MAPbI_3_ structure, equilibrated by performing NPT simulations at 330 K. A ball-and-stick view of the unit cells for perfect (left-top panel) and defective crystalline solids containing vacancies such as an MA vacancy (right-top), Pb vacancy (left-bottom) and I vacancy (right-bottom), with average bond lengths of *L*_Pb−Pb_ denoted by red-colored arrows and *L*_I−I_ by blue-colored arrows. Color legends are dark gray for Pb, pink for I, purple for N, brown for C and white for H.

For the EMD simulations, we created 8 × 8 × 8 supercells with 6144 atoms for the pseudocubic phase (Fig. 2) and 5 × 5 × 5 supercells with 6000 atoms for the tetragonal phase of perfect MAPbI_3_ (Fig. S1†). To create defective MAPbI_3_ structures, we remove a certain number of molecular moieties or atoms in the 8 × 8 × 8 supercells. With respect to the defect concentration, Walsh *et al.*^26^ pointed out that the concentration of vacancy defects, such as the MA vacancy V_MA_, Pb vacancy V_Pb_ and I vacancy V_I_, should exceed 0.4% in cubic MAPbI_3_ at room temperature, based on their DFT calculations. Accordingly, the vacancy concentrations in the defective MAPbI_3_ were allowed to vary from 0.0 to 1.0% with an interval of about 0.2% in this work. The simulation cells for defective systems with these vacancy concentrations were created by randomly removing MA^+^ and Pb^2+^ cations with numbers from 1 to 5 with an interval of 1 and I^−^ anions with numbers from 6 to 15 with an interval of 3. After removing the ions, we introduced a uniform background charge to neutralize the defective charged MAPbI_3_ system as applied in the previous MD simulations.^37^

For the AEMD simulations, we adopted the perfect tetragonal phase with simulation supercells with cell lengths of *L*_*x*_ = *L*_*y*_ ≃ 2.6 nm and *L*_*z*_ ≃ 38–152 nm (see Fig. S2† for *L*_*z*_ ≃ 51 nm). In fact, it was confirmed from the previous work^20^ that *κ* calculated with *L*_*x*_ = *L*_*y*_ ≃ 3 nm was changed by only 5% from that with *L*_*x*_ = *L*_*y*_ ≃ 11 nm.

All the MD simulations were performed using the LAMMPS package.^38^ The *pppm* solver with an accuracy of 10^−5^ was used to compute the coulombic interactions, and the velocity Verlet algorithm was used for integrating motion with a time step of 0.5 fs. Periodic boundary conditions were applied in the *x*, *y* and *z* directions. The control of temperature and pressure during the simulation was managed by the Nosé–Hoover thermostat and barostat. In the AEMD simulations, the entire system was initially relaxed by NPT simulations at a temperature of 300 K and pressure of 0 atm for 500 ps. Then, two split regions 1 and 2 were equilibrated by NVT simulations at different temperatures of 325 K and 275 K for 500 ps. Subsequently, NVE equilibration was carried out for sufficient simulation time, which was different depending on the cell length. In the EMD simulations, meanwhile, NVE simulation was carried out for 5 ns after NPT and subsequently NVT equilibrations for 500 ps. To reduce statistical error, we repeated 5 and 10 independent runs with different initial atomic velocities for both perfect and defective models. For calculations of elastic constants, we performed molecular mechanic (MM) simulations after NPT equilibration at 330 K for 10^6^ steps.

### 2.3 MYP force field

Choosing a suitable force field is a very important factor for determining the accuracy of classical MD simulations. To date, several force fields have been developed for MAPbI_3_,^18,19,39–42^ and among them the MYP force field derived from *ab initio* DFT calculations^39,40^ has been widely used to study the lattice thermal properties of MAPbI_3_.^17,20^ In particular, Barboni *et al.* calculated the jumping rate of iodine vacancies in defective MAPbI_3_ using the MYP force field.^37^ In this work, we also employed the MYP force field to investigate the effect of vacancy defects on the lattice thermal properties of MAPbI_3_.

The MYP force field is composed of the three terms,

4 *U*_Total_ = *U*_OO_ + *U*_II_ + *U*_OI_

where *U*_OO_, *U*_II_ and *U*_OI_ are the organic–organic, inorganic–inorganic and organic–inorganic potentials, respectively. Thus, the *U*_OO_ part contains intermolecular and intramolecular interactions between the MA moieties as described in the standard AMBER force field,^43^ and the *U*_II_ part includes the interaction between Pb and I atoms with the Buckingham-type potential. Meanwhile, the *U*_OI_ part is the sum of Buckingham, coulombic and Lennard-Jones (12-6) terms as follows,^39^5
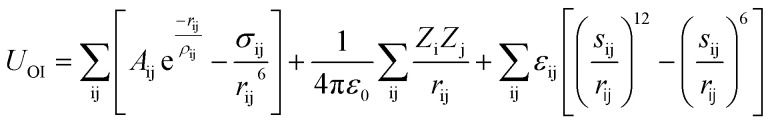
where *r*_ij_ is the distance between the i-th and j-th atoms, *Z*_i_ is the partial charge of the i-th atom, *ε*_0_ is the dielectric constant of a vacuum, and *ρ*_ij_, *σ*_ij_, *ε*_ij_, *s*_ij_ are the empirical parameters. These potential parameters are provided in Tables S1–S6 (see the ESI†) and can be found in the previous work.^39^

## 3 Results and discussion

### 3.1 Thermal conductivity of a perfect system

To verify the accuracy of the selected methods, we calculated the thermal conductivity of perfect MAPbI_3_ at 300 K using both the AEMD and EMD methods, comparing with the previous theoretical and experimental data. Firstly, the AEMD method was applied to calculate the *κ* value of perfect MAPbI_3_ with a tetragonal structure at a temperature of 300 K. Through the analysis of NPT simulation, we found that the equilibrated lattice parameters and elastic properties of the tetragonal phase agreed well with the previous data (Table S7†).

It was known that the *κ* value of an insulating solid, calculated using MD simulations, depends on the size of the simulation box when the size is smaller than the mean free path of the phonon.^20^ The dependence of *κ* on the simulation box size can be expressed as,6
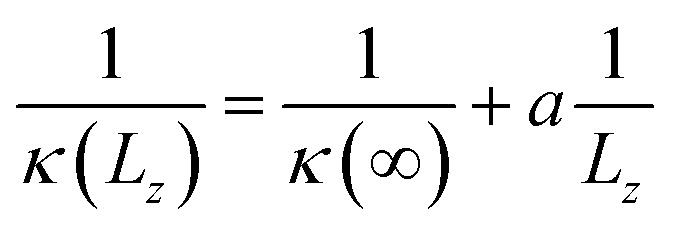
where *κ*(*L*_*z*_) and *κ*(∞) are the thermal conductivities of samples with finite and actual size, respectively, and *a* is the fitting parameter. This indicates that the actual *κ*(∞) can be obtained by applying linear extrapolation of 1/*κ vs.* 1/*L*_*z*_.

To estimate *κ*(*L*_*z*_) with increasing *L*_*z*_, we need to determine the volumetric heat capacity *C*_V_ and mass density *ρ*. The heat capacity was estimated using the linear relation between the total energy *E* and temperature *T* as 
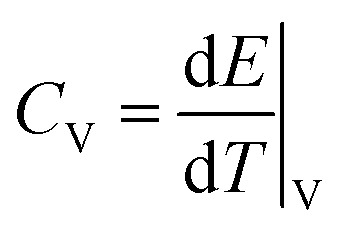
. Linear fitting of the total energy *E* of the system at different temperatures (see Fig. S3†) produced *C*_V_ as 1.327 × 10^−5^ eV K^−1^ Å^−3^ in good agreement with the previous value of about 1.3 × 10^−5^ eV K^−1^ Å^−3^.^20^

**Fig. 3:**
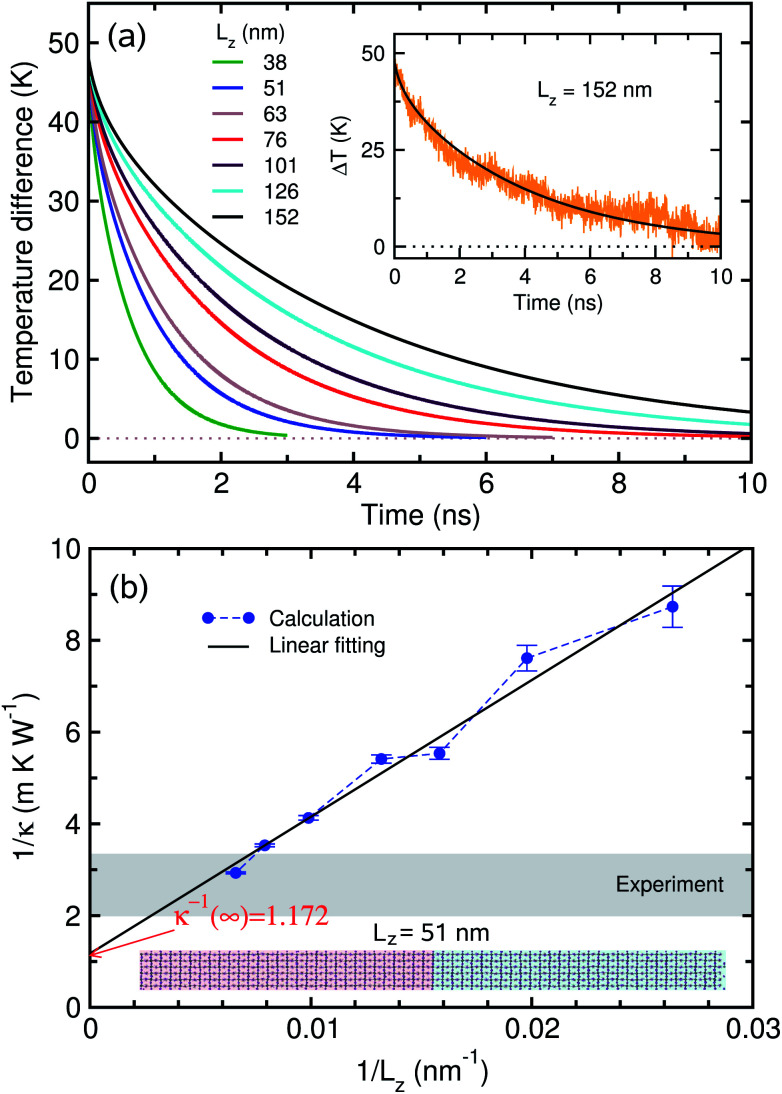
(a) The average temperature difference Δ*T* as simulation time progresses, with increasing simulation box size *L*_*z*_, fitted to an exponential function. The inset shows Δ*T* as a function of simulation time for real values (orange-colored line) and the fitting line (black-colored smooth line) for the case of *L*_*z*_ = 152 nm. (b) The calculated inverse of thermal conductivity 1/*κ* with different sizes *L*_*z*_, and linear extrapolation of 1/*κ vs.* 1/*L*_*z*_.

In the AEMD simulation, it was found that as the NVE simulation proceeds, the average temperature difference Δ*T* between the two sub-regions decreases, finally becoming the final value of zero, as shown in Fig. 3(a). Initially, Δ*T* was 50 K for all cases of cell size *L*_*z*_, which were set from 38 nm to 152 nm with 5 intermediate points. While increasing the cell size *L*_*z*_, the decreasing rate of Δ*T* gets slower while the fitting accuracy increases. In each case of *L*_*z*_, we performed fitting of the obtained Δ*T*(*t*) (orange line shown in the inset of Fig. 3(a)) to an exponential function (see the ESI for details†), yielding the thermal diffusivity *κ̄* and thus the thermal conductivity *κ*(*L*_*z*_). We then carried out linear fitting of 1/*κ*(*L*_*z*_) *vs.* 1/*L*_*z*_, as shown in Fig. 3(b), and thus obtained 1/*κ*(∞) as 1.172 m K W^−1^. This gives the actual *κ* of tetragonal MAPbI_3_ as 0.853 W m^−1^ K^−1^, which is in good agreement with the previous simulation data of 0.8 W m^−1^ K^−1^ obtained using the same MYP force field and the same AEMD method.^20^

**Fig. 4:**
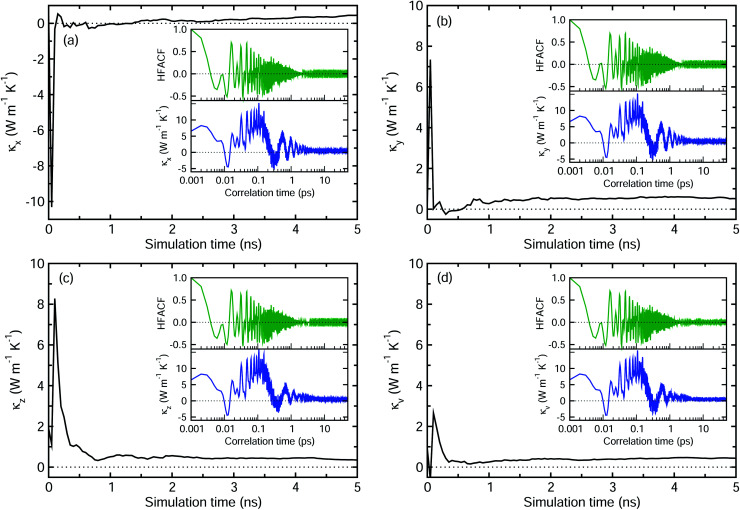
Principal thermal conductivity in each Cartesian direction, including *κ*_*x*_ (a), *κ*_*y*_ (b) and *κ*_*z*_ (c), and the volumetric thermal conductivity *κ*_v_ (d) as their average for perfect MAPbI_3_. After a simulation time of 4 ns, the thermal conductivity is found to converge well to a certain value. The insets show the heat flux auto-correlation function (HFACF) and lattice thermal conductivity as a function of correlation time, which are found to converge well to zero and a certain value after 10 ps.

Next, we applied the EMD method, *i.e.*, the Green–Kubo method, to calculate the *κ* value of perfect MAPbI_3_. Fig. 4 shows the principal *κ*_*α*_ in each Cartesian direction (*α* = *x*, *y*, *z*) and the volumetric *κ*_v_ for perfect MAPbI_3_ as the EMD simulation proceeds, these were dumped every 50 ps to estimate the HFACF and *κ vs.* correlation time (see insets). The HFACF and *κ* were plotted every 1 fs, and they converged to zero and a certain value after 10 ps, respectively. The principal thermal conductivities were determined to be *κ*_*x*_ = 0.328, *κ*_*y*_ = 0.480 and *κ*_*z*_ = 0.406 W m^−1^ K^−1^, and their average was determined to be *κ*_v_ = 0.405 W m^−1^ K^−1^. These agree well with the previous simulation data of 0.25–0.40 W m^−1^ K^−1^, obtained using the same MYP atomic potential and the same EMD method,^17^ and the experimental values of 0.3–0.5 W m^−1^ K^−1^ for perfect MAPbI_3_.^5,11,13^ It is worth noting that the anisotropy of the thermal conductivity is associated with the structural anisotropy of the MAPbI_3_ crystal with the orientation of the MA cation.^10^

Although the same interatomic potential was used, the AEMD and EMD methods produced different thermal conductivities. In principle, these two methods should give similar results. It seems that the AEMD method gives the upper limit of thermal conductivity for the bulk, since it refers to an infinitely long sample with perfect crystallinity. In Fig. 3(b), the shaded region indicates the experimental range, for which *L*_*z*_ is between 130 nm and 330 nm, being considered as the “effective” mean free path in accordance with the experimental coherence length below 150 nm.^44^ The AEMD method has a much larger computational burden for a solid with low *κ* like MAPbI_3_ because Δ*T* diminishes more slowly to zero. Therefore, the EMD method is used for defective MAPbI_3_.

**Fig. 5:**
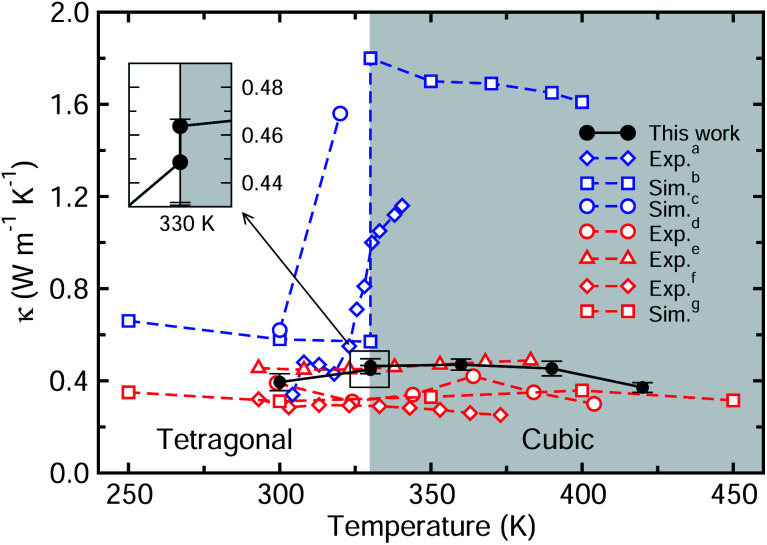
Thermal conductivity of perfect MAPbI_3_ as a function of temperature. The inset shows two points for tetragonal and pseudocubic phases at 330 K. Blue-colored dotted lines represent the previous data (exp.^a^ (ref. 13), sim.^b^ (ref. 18) and sim.^c^ ref. 15)) with a rapid jump around the phase transition temperature of 330 K, while red-colored dotted lines show the data without a jump (exp.^d^ (ref. 5), exp.^e^ (ref. 6), exp.^f^ (ref. 14) and sim.^g^ (ref. 17)).

We then investigate the temperature dependence of lattice thermal conductivity of perfect MAPbI_3_ as the temperature increases from 300 to 420 K with an interval of 30 K. Fig. 5 shows *κ* as a function of temperature calculated with the EMD method in comparison with the previous theoretical and available experimental data. In our work, there is a minor difference of about 0.015 W m^−1^ K^−1^ between the tetragonal (0.449 W m^−1^ K^−1^) and pseudocubic (0.464 W m^−1^ K^−1^) phases at 330 K. Some previous work reported a big jump in the thermal conductivity at 330 K upon phase transition from the tetragonal to pseudocubic phase (blue dotted lines),^13,15,18^ whereas more recent works revealed that *κ* does not abruptly increase at 330 K, even upon phase transition (red dotted lines).^5,6,14,17,45^ Our work agrees with the latter cases, confirming that the lattice thermal conductivity rarely changes upon increasing temperature. Qian *et al.*^18^ adopted different interatomic potentials for different phases, giving a big jump in thermal conductivity upon phase transition from tetragonal to cubic phases, but this makes it difficult to associate the thermal conductivity with the cation dynamics. Meanwhile, Wang *et al.*^17^ used the same MYP potential for different phases, resulting in no jump in thermal conductivity as in our work. This is connected with the viewpoint that the MA cation has a minor effect on the thermal transport at higher temperatures, since the difference in crystal structures between tetragonal and cubic phases is in the distribution and orientation of the MA cations.

### 3.2 Thermal conductivity of defective system

**Fig. 6:**
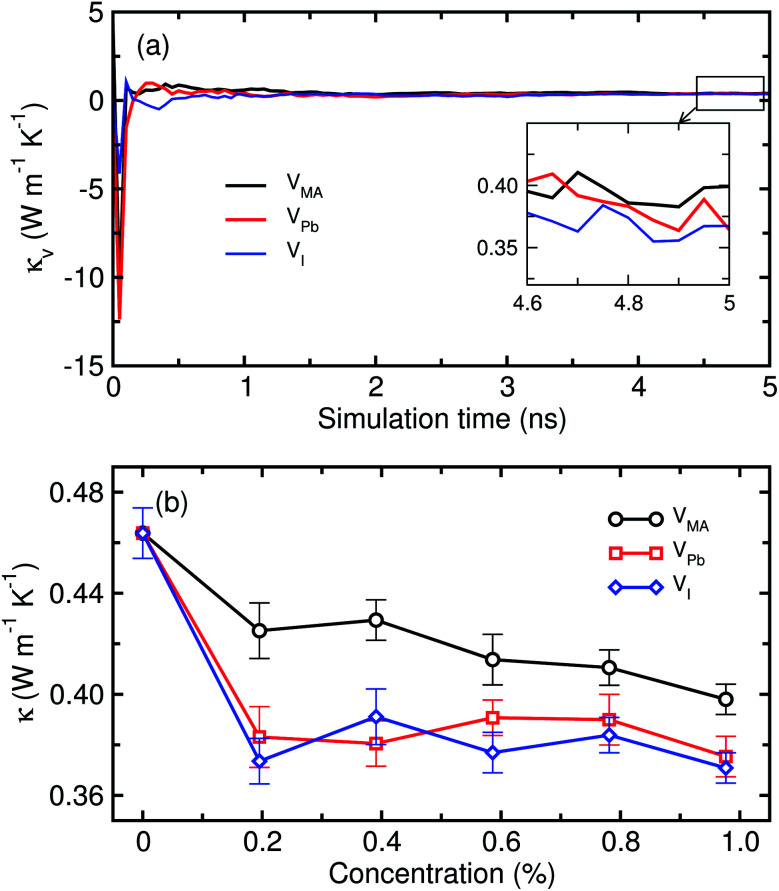
Thermal conductivity of defective MAPbI_3_ in the pseudocubic phase as a function of (a) simulation time and (b) vacancy concentration – MA vacancy V_MA_, Pb vacancy V_Pb_ and I vacancy V_I_.

Next, we calculated the lattice thermal conductivities of the defective MAPbI_3_ systems in the pseudocubic phase at 330 K, which contains three kinds of vacancies, V_MA_, V_Pb_ and V_I_, with different concentrations ranging from 0.0 to 1.0% with an interval of 0.2%. Fig. 6 shows the calculated *κ* values of defective MAPbI_3_ as a function of vacancy concentration. It was found that the thermal conductivity decreases gradually when creating the vacancy defects, and shows a decreasing tendency overall, as the vacancy concentration increases. At the beginning of vacancy creation, between 0 and 0.2% concentration, the reductions in *κ* for V_I_ and V_Pb_ were large compared to those for the case of V_MA_. On the way to 1%, although there are some slight rises at 0.4% for V_MA_ and V_I_, and at 0.6 and 0.8% for V_Pb_, we could say that the thermal conductivities of defective systems decrease overall as the concentration increases from 0% to 1%. These slight rises at the middle concentrations may come from the intrinsic fluctuation feature of classical MD simulations, although we repeated the simulations at least 10 times. For comparison among the kinds of vacancy, V_I_ causes the greatest reduction of *κ* from 0.464 ± 0.011 W m^−1^ K^−1^ at 0% to 0.371 ± 0.006 W m^−1^ K^−1^ at 1%, V_Pb_ shows the next greatest reduction to 0.375 ± 0.008 W m^−1^ K^−1^ at 1%, and V_MA_ gives the least reduction to 0.398 ± 0.006 W m^−1^ K^−1^ at 1% concentration. In general, it can be thought that the creation of vacancy defects in an insulating crystalline solid causes a disturbance of the (acoustic) phonon propagation, reducing the lattice thermal conductivity as these are the main thermal carriers. From our calculation results, it can be regarded that the atomic vacancies of V_I_ and V_Pb_ have a greater scattering effect on phonon transfer than the molecular vacancy of V_MA_. This is related to the fact that the acoustic phonon modes carrying the heat are mainly from the Pb–I cage, while the MA molecule plays a role in scattering phonons.

For each case of vacancy defects, we discuss the mechanism behind the reduction of lattice thermal conductivity of MAPbI_3_ in comparison with the previous theoretical and experimental data. In the case of V_MA_, it should be noted that the organic MA^+^ cations are the main contributors to the ultralow thermal conductivity of MAPbI_3_.^11,15,17,19,20^ It was found that coupling between the rotational and translational motions of the MA cation plays a key role in suppressing the thermal conductivity of MAPbI_3_.^19^ In fact, Hata *et al.* demonstrated through MD simulations that the real MAPbI_3_ clearly exhibits a lower *κ* value (0.185 W m^−1^ K^−1^) compared to the hypothetical PbI_3_ bare inorganic frame model (0.326 W m^−1^ K^−1^), finding that the rotational motions of MA cations mainly induce the suppression of thermal conductivity.^19^ Moreover, coupled rotational and translational motions of MA were found to interact with the Pb–I cages and derive couplings between the lattice vibrations, suppressing the phonon transport in MAPbI_3_. The removal of some molecules leads to a lower number of molecular degrees of freedom and thus could favor the thermal transport.^20^ According to our calculations, however, *κ* decreases as the concentration of MA vacancies increases, in contrast to the previous data and general insight. The MA^+^ cation seems to have a significant suppressing effect on *κ* only at low temperature, while it has a relatively small contribution at room temperature.^12^ Instead, the internal phonon–phonon scattering dominantly influences the thermal transport in halide perovskites at room temperature. In fact, similar ultralow thermal conductivities were observed in the all-inorganic halide perovskites with no organic molecule such as 0.36 W m^−1^ K^−1^ for single crystalline nanowire CsPbBr_3_ (ref. 46) and 0.45 ± 0.05 W m^−1^ K^−1^ for CsPbI_3_ (ref. 47) at room temperature, being associated with the metal-halide cluster rattling modes. From such considerations, it can be noticed that, at higher temperature, the MA^+^ cation contributes negligibly to the lattice thermal conductivity of MAPbI_3_. The MA molecular vacancies associated with the local perturbations of the PbI charge and structure can act as scattering centers for heat transfer and induce Rayleigh-like scattering of phonons, resulting in a reduction of thermal conductivity of MAPbI_3_.^20^

For the case of Pb vacancies, one can see that *κ* decreases more rapidly than in the V_MA_ case as the vacancy concentration increases, indicating that the effect of V_Pb_ on *κ* is more pronounced than that of V_MA_. Considering that the acoustic phonon modes are dominated by Pb and I atoms, it is natural to see such reduction of *κ* by the creation of V_Pb_, which can play the role of phonon scattering center. Neither theoretical nor experimental data for this V_Pb_ effect was reported before. Here, it is worth noting that when substituting a lighter metal cation for Pb, the lattice thermal conductivity of HPs was found to decrease for the pseudocubic phase but increase for the tetragonal phase. For instance, upon replacing Pb with Sn in MAPbI_3_, Mettan *et al.* found a decrease of *κ* from 0.5 W m^−1^ K^−1^ to 0.09 W m^−1^ K^−1^ for the pseudocubic phase, but an increase to 0.69 W m^−1^ K^−1^ for the tetragonal phase.^51^ In the case of all-inorganic halide perovskites, Yang *et al.* revealed a decrease from the 0.45 ± 0.05 W m^−1^ K^−1^ of CsPbI_3_ to 0.38 ± 0.04 W m^−1^ K^−1^ for CsSnI_3_.^47^ The *κ* reduction upon this replacement is because the third order force constant in the Sn–I bond is larger than that in the Pb–I bond, resulting in stronger anharmonicity.^52^

The role of the iodine vacancy is similar to the case of V_Pb_ as shown in Fig. 6, presenting that *κ* rapidly decreases as the V_I_ concentration increases. We discuss the effect of substituting a lighter halogen anion for the I^−^ anion on the thermal conductivity. From experiments, the thermal conductivity of single crystalline MAPbX_3_ solid was found to increase from 0.34 ± 0.12 W m^−1^ K^−1^ for X = I to 0.44 ± 0.08 W m^−1^ K^−1^ for X = Br and to 0.50 ± 0.05 W m^−1^ K^−1^ for X = Cl.^13^ A similar tendency was observed for the cases of all-inorganic halide perovskites.^53^ Such an increase in thermal conductivity with decreasing the atomic number of the halogen atom is associated with the increasing elastic stiffness constants.

Our simulation result of the thermal conductivity of defective MAPbI_3_, containing vacancies such as V_MA_, V_Pb_ and V_I_, indicates that the low thermal conductivity of MAPbI_3_ originates from the inorganic Pb–I framework lattice.^14,19,20^ From this perspective, it is worth noting that the *κ* value of the PbI_2_ solid is also very low, 0.22 W m^−1^ K^−1^ at 293 K.^14^ In many previous studies of HPs, it has been found that a heavier mass of halide atom could lead to lower thermal conductivity. If the constituent atoms are heavier, *i.e.*, with larger atomic number and larger ionic radius, the phonon vibrations in principle get weaker while strengthening its scattering, thereby reducing thermal transport. In particular, the electronegativity of the X atom decreases with increasing the atomic mass, and thus the B–X bond length increases, resulting in lowering the thermal conductivity. For the case of the B-site atom, there are only a few studies for thermal conductivity, and therefore, more work is needed to elucidate the effect of changing the B-site metal.^10^ Such big effects of the B-site metal and X-site halogen can provide the possibility of tuning the thermal conductivity of MA-related HPs, MABX_3_.

### 3.3 Elastic properties of perfect and defective systems

Many previous studies analyzed the thermal transport properties of MAPbI_3_ using the phonon group velocity and phonon lifetime based on a picture of particle-like phonon propagation in crystals. However, recent studies have proposed a coexistence of crystal- and glass-like transport mechanisms for heat conduction in HPs, demonstrating that the crystal-like transport mechanism is dominant at low temperatures while the glass-like transport plays a dominant role at higher temperatures.^54^ For the case of CsPbBr_3_, for instance, crystal- and glass-like contributions to *κ* were found to be 78% and 22% at 50 K, while 30% and 70% at 300 K.^54^ This indicates that description of *κ* only by particle-like phonon propagation is not adequate for HPs at room temperature.

For the description of crystal- and glass-like thermal transport, the elastic moduli and sound velocity are interesting because they are evaluated for polycrystalline solids. In fact, Elbaz *et al.* scaled the thermal conductivity with the sound velocity to explain the measured low thermal conductivities of 0.34–0.73 W m^−1^ K^−1^ at room temperature for MAPbX_3_ (X = I, Br, Cl).^50^ Moreover, the order of thermal conductivities of HPs was determined from the mechanical properties and sound velocities.^17^ Therefore, we evaluated the mechanical properties, including elastic constants and moduli, and the sound velocity from them for perfect and defective MAPbI_3_, this can be meaningful in explaining the variation tendency of *κ* upon the creation of vacancies.

**Fig. 7:**
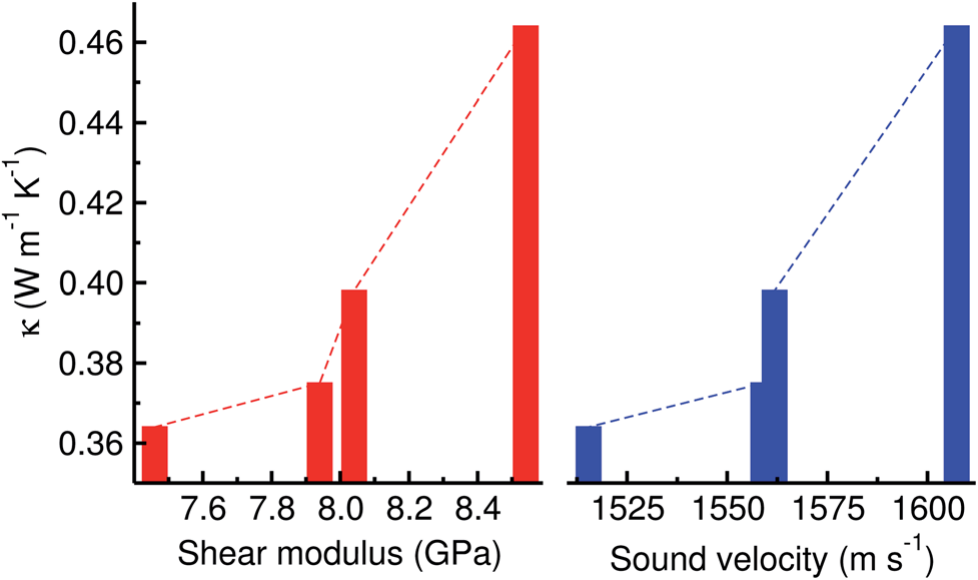
Thermal conductivity *vs.* shear modulus and sound velocity for defective MAPbI_3_. Dashed lines are to guide the eye.

Table 1 presents the calculated elastic stiffness constants (*C*_*ij*_), elastic moduli including bulk (*B*), shear (*G*) and Young’s (*E*) moduli, Poisson’s ratio (*ν*), density (*ρ*), and sound velocity (*v*), with the thermal conductivity (*κ*) for perfect and defective MAPbI_3_ in the pseudocubic phase at a vacancy concentration of 1.0%. Our results from the classical EMD method were found to be in good agreement with the previous calculations from the DFT method. It should be noted that, although the pseudocubic phase was initially assigned, the final phase equilibrated by NPT simulation was found to be orthorhombic with very small differences of lattice constants due to the numerical noise being inherent in MD simulation, leading to 9 independent elastic constants. We should point out that the average sound velocity of 1922 m s^−1^ by Faghihnasiri *et al.*^49^ was evaluated with a mistaken equation and thus we corrected it to be 1628.6 m s^−1^ by applying the correct equation (eqn (S14)†), which is in good agreement with our results and other calculations.^48^ It was found that the elastic constants, moduli and sound velocities of defective MAPbI_3_ are smaller than those of the perfect system, leading to the lower thermal conductivity. As shown in Fig. 7 and Table 1, there is a certain relation between the thermal conductivity and elastic modulus or sound velocity; larger elastic constants and sound velocities lead to higher thermal conductivity.

**Table 1:** Elastic stiffness constant (*C*_*ij*_), bulk modulus (*B*), shear modulus (*G*), Young’s modulus (*E*), Poisson’s ratio (*ν*), mass density (*ρ*), average sound velocity (*v*) and thermal conductivity (*κ*) for perfect and defective MAPbI_3_

System	Elastic stiffness constants (GPa)									Elastic moduli (GPa)			*ν*	*ρ* (g cm^−3^)	*v* (m s^−1^)	*κ* (W m^−1^ K^−1^)
	*C*_11_	*C*_12_	*C*_13_	*C*_22_	*C*_23_	*C*_33_	*C*_44_	*C*_55_	*C*_66_	*B*	*G*	*E*				
Perfect	24.4	13.5	12.4	25.9	12.6	23.8	10.5	10.6	11.9	16.74	8.54	21.89	0.282	4.106	1607.3	0.464
Perfect^a^	27.1	11.1					9.2			16.40	8.70	22.20	0.280		1620.0	
Perfect^b^	35.4	10.0					6.1			18.50	8.70	22.80	0.220		1922.0 (1628.6)	
Perfect^c^												12.00		4.119	1390.0	0.340
V_MA_	23.5	12.9	11.9	26.1	12.7	20.3	10.7	10.3	11.0	15.93	8.04	20.70	0.284	4.096	1561.8	0.398
V_Pb_	23.4	12.2	12.9	24.1	11.8	21.4	10.3	10.8	10.4	15.82	7.94	20.41	0.285	4.060	1559.0	0.375
V_I_	22.9	13.0	12.7	24.1	12.4	20.0	10.1	10.3	10.0	15.85	7.46	19.35	0.296	4.049	1515.4	0.364

a DFT calculation data with the PBE functional.^48^

b DFT calculation data with the PBEsol + vdW functional.^49^

c Experimental data for the tetragonal structure.^50^

## 4 Conclusions

In this work, we have investigated the lattice thermal conductivity of perfect and defective MAPbI_3_ using classical MD simulations with the MYP force field. With the AEMD and EMD methods, the thermal conductivity of perfect MAPbI_3_ was determined to be 0.853 and 0.405 W m^−1^ K^−1^ at 300 K using the supercells, and its temperature dependence was studied by increasing the temperature from 300 K to 420 K with the EMD method. We made supercell models of defective systems containing vacancy defects such as V_MA_, V_Pb_ and V_I_ with different concentrations to investigate the effect of vacancy concentration on the thermal conductivity. Our calculations revealed that, as the vacancy concentration increases from 0 to 1% with an interval of 0.2%, the thermal conductivity decreases overall with slight rises at 0.4% for V_MA_ and V_I_ and at 0.6 and 0.8% for V_Pb_. We have shown that such vacancies can act as phonon scattering centers, thereby causing the decrease in thermal conductivity. Furthermore, the elastic moduli and sound velocities of defective MAPbI_3_ were determined to be smaller than those of the perfect system, revealing that the low sound velocity in relation with the bulk and shear moduli is responsible for the ultralow thermal conductivity of MAPbI_3_. Our results are helpful for designing functional hybrid halide perovskites for photovoltaic and thermoelectric devices with high performance.

## Author contributions

Song-Nam Hong and Chol-Jun Yu developed and planned the original project. Song-Nam Hong performed the calculations and drafted the first manuscript. Chol-Jun Yu supervised the work. Song-Hyok Choe assisted with the MD simulation. Un-Gi Jong and Yun-Hyok Kye contributed to useful discussions. All authors reviewed the manuscript.

## Conflicts of interest

There are no conflicts to declare.

## Supplementary Material

RA-011-D1RA05393K-s001
