# Supplementary material for: Effect of vacancy concentration on the lattice thermal conductivity of CH3NH3PbI3: a molecular dynamics study
Source: RSC Adv. 2021 Oct 20;11(54):34015–23. doi: 10.1039/d1ra05393k (PMC9042382; doi:10.1039/d1ra05393k)
Supplement: RA-011-D1RA05393K-s001 [file RA-011-D1RA05393K-s001.pdf]

Electronic Supplementary Material (ESI) for RSC Advances.
This journal is © The Royal Society of Chemistry 2021

Supplemental Material – Effect of vacancy concentration on lattice thermal conductivity of $CH_3NH_3PbI_3$: A molecular dynamics study

Song-Nam Hong[1], Chol-Jun Yu[1],* Un-Gi Jong[1,2], Song-Hyok Choe[1,2], Yun-Hyok Kye[1,2]

[1]*Chair of Computational Materials Design (CMD), Faculty of Materials Science, Kim Il Sung University, Pyongyang, PO Box 76, Democratic People's Republic of Korea*

[2]*Natural Science Center, Kim Il Sung University, Pyongyang, PO Box 76, Democratic People's Republic of Korea*

AEMD method

In the AEMD method, the two sub-regions are initially equilibrated with periodic boundary condition at different temperatures ($T_1 > T_2$),

$$T(z) = \begin{cases} T_1 & \text{for} \quad 0 < z \leq L_z/2 \\ T_2 & \text{for} \quad L_z/2 < z \leq L_z \end{cases}$$

where L_z is the length of the system in z-direction. Then, the temperature difference can be expanded by using exponential function as follows [1],

$$\Delta T(t) = \langle T_1(t) \rangle - \langle T_2(t) \rangle = \sum_{n=1}^{\infty} C_n e^{-\alpha_n^2 \bar{\kappa} t} \tag{S1}$$

where $\langle T_1(t) \rangle$ and $\langle T_2(t) \rangle$ are the average temperatures of sub-regions 1 and 2 above time t, $\alpha_n = 2\pi n/L_z$, and $\bar{\kappa}$ is the fitting parameter. The expansion coefficients C_n can be written as follows,

$$C_n = 8(T_1 - T_2) \frac{[\cos(\alpha_n L_z/2) - 1]^2}{\alpha_n^2 L_z^2} = 8(T_1 - T_2) \frac{\left[\cos(\frac{2\pi}{L_z} n \frac{L_z}{2}) - 1\right]^2}{\left(\frac{2\pi}{L_z} n L_z\right)^2} = \frac{8(T_1 - T_2)}{(2\pi)^2} \frac{[\cos(n\pi) - 1]^2}{n^2} \tag{S2}$$

Then, the equation S1 can be expanded as follows,

$$\Delta T(t) = \frac{8(T_1 - T_2)}{(2\pi)^2} \left\{ \frac{(\cos\pi - 1)^2}{1^2} e^{-\left(\frac{2\pi}{L_z}\right)^2 \bar{\kappa} t} + \frac{(\cos 3\pi - 1)^2}{3^2} e^{-9\left(\frac{2\pi}{L_z}\right)^2 \bar{\kappa} t} + \frac{(\cos 5\pi - 1)^2}{5^2} e^{-25\left(\frac{2\pi}{L_z}\right)^2 \bar{\kappa} t} + \cdots \right\} \tag{S3}$$

Note that the even number terms are zero and thus removed from the expansion. For the sake of simplicity, the fitting parameter τ_0 is introduced instead of $\bar{\kappa}$ using the following relation,

$$\left(\frac{2\pi}{L_z}\right)^2 \bar{\kappa} = \frac{1}{\tau_0} \tag{S4}$$

Then, the above equation can be written as follows,

$$\Delta T(t) = \frac{8(T_1 - T_2)}{\pi^2} \left\{ e^{-\frac{t}{\tau_0}} + \frac{1}{9} e^{-9\frac{t}{\tau_0}} + \frac{1}{25} e^{-25\frac{t}{\tau_0}} + \cdots \right\} = \frac{8(T_1 - T_2)}{\pi^2} \sum_{n=1}^{\infty} (2n-1)^{-2} \exp\left[\frac{-(2n-1)^2}{\tau_0} t\right] \tag{S5}$$

Note that $n = 5$ can give the sufficient accurate fitting result. Then, the thermal conductivity κ can be determined by using the following equation,

$$\kappa = \bar{\kappa} \cdot \rho C_V = \frac{L_z}{(2\pi)^2} \frac{\rho C_V}{\tau_0} \tag{S6}$$

where ρ is the density and C_V is the volumetric heat capacity.

*Corresponding author: Chol-Jun Yu, Email: cj.yu@ryongnamsan.edu.kp

Mechanical properties from elastic constants

The lower limits of bulk and shear moduli within the Voigt approximation can be calculated using the stiffness elast constants as follows,

$$B_V = [C_{11} + C_{22} + C_{33} + 2(C_{12} + C_{13} + C_{23})] /9 \tag{S7}$$

$$G_V = [C_{11} + C_{22} + C_{33} + 3(C_{44} + C_{55} + C_{66}) - (C_{12} + C_{13} + C_{23})] /15 \tag{S8}$$

The upper limits of bulk and shear moduli can be calculated within the Reuss approximation as follows,

$$B_R = [S_{11} + S_{22} + S_{33} + 2(S_{12} + S_{13} + S_{23})]^{-1} \tag{S9}$$

$$G_R = 15[4(S_{11} + S_{22} + S_{33} - S_{12} - S_{13} - S_{23}) + 3(S_{44} + S_{55} + S_{66})]^{-1} \tag{S10}$$

Mechanical stability of a polycrystalline solid can be assessed by calculating the elastic constants and elastic moduli such as bulk modulus (B), shear modulus (G) and Young's modulus (E). According to the Voigt-Reuss-Hill approximation, the bulk and shear moduli of a polycrystalline solid can be determined as follows,

$$B = \frac{B_V + B_R}{2}, \ G = \frac{G_V + G_R}{2} \tag{S11}$$

where B_V and G_V (B_R and G_R) are the upper (lower) limits of the polycrystalline bulk and shear moduli within the Voigt (Reuss) approximation. These moduli can be estimated by using the elastic stiffness and compliance tensors that can be readily obtained from MD simulation. From these moduli, the Young's modulus E and Poisson ratio ν are estimated as follows,

$$E = \frac{9GB}{3B + G}, \ \nu = \frac{3B - 2G}{6B + 2G} \tag{S12}$$

Given these elastic properties, one can estimate the sound speed, which is closely connected with the thermal properties. The transverse (v_t) and longitudinal (v_l) sound velocities are given as follows,

$$v_t = \sqrt{\frac{G}{\rho}}, \ v_l = \sqrt{\frac{3B + 4G}{3\rho}} \tag{S13}$$

Finally, the average sound velocity is estimated using these transverse and longitudinal sound velocities as follows,

$$v = \left[\frac{1}{3}\left(\frac{2}{v_t^3} + \frac{1}{v_l^3}\right)\right]^{-1/3} \tag{S14}$$

Table S1. Parameters for Buckingham potential

	A (eV)	ρ (Å)	C (Å^6)
I-I	989.0255841	0.482217	30.24133
Pb-I	4490.799934	0.321737	0.0
Pb-Pb	3052986.18284579	0.131258	0.0
Pb-C	1418468.50893	0.1507947	0.0
Pb-N	1418468.50893	0.1507947	0.0
I-C	4900.4361	0.342426	0.0
I-N	4900.4361	0.342426	0.0

Table S2. Parameters for L-J potential

	A (eV)	ρ (Å)
Pb-h+	0.000607	2.26454
Pb-hc	0.000607	2.70999
I-h+	0.00249	2.75
I-h+	0.00249	3.10
N-C	0.00592	3.3248
C-C	0.004747	3.3997
N-N	0.0073765	3.25
N-h+	0.0022432	2.15950
N-hc	0.0022432	2.6050
h+-h+	0.0006812	1.0691
h+-hc	0.0006812	1.5145
hc-hc	0.0006812	1.9600
h+-C	0.0017964	2.2344
hc-C	0.0017964	2.6798

”h+” and ”hc” denote hydrogen connected to N and C atoms.

Table S3. Parameters of bond potential

	α_i (eV/Å^2)	$r_{i,equ}$ (Å)
N-h+	32.021353	1.033
C-hc	29.391957	1.091
C-N	25.478237	1.499

Table S4. Parameters for angle potential

	β_i (eV/rad^2)	$\theta_{i,equ}$ (deg)
h+-N-h+	3.51454	108.11
C-N-h+	4.00918	110.11
N-C-hc	4.25216	107.91
hc-C-hc	3.38437	110.74

Table S5. The parameters of dihedral potential

	K (eV)	N	d (deg)	Weighting factor
hc-C-N-h+	0.006751	3	0	0.0

Table S6. Masses and electric charges for various atoms

	m_i (g/mol)	q_i (e)
h+	1.008	0.54
hc	1.008	0.023
N	14.01	−1.1
C	12.01	0.771
Pb	207.2	2.03
I	126.9	−1.13

Table S7. Lattice parameters and elastic properties of bulk modulus B, shear modulus G, Young's modulus E and Poisson's ratio ν for $MAPbI_3$ with tetragonal structure at 300 K, together with the previous DFT calculation and available experimental data.

	a (Å)	b (Å)	c (Å)	B (GPa)	G (GPa)	E (GPa)	ν
This work	8.771	8.791	12.946	18.9	5.9	16.0	0.36
DFT [2]	8.800	8.800	-	12.2	3.7	12.8	0.33
Exp [3]	8.849	8.849	12.642	13.9	5.4	14.0~14.3	0.33

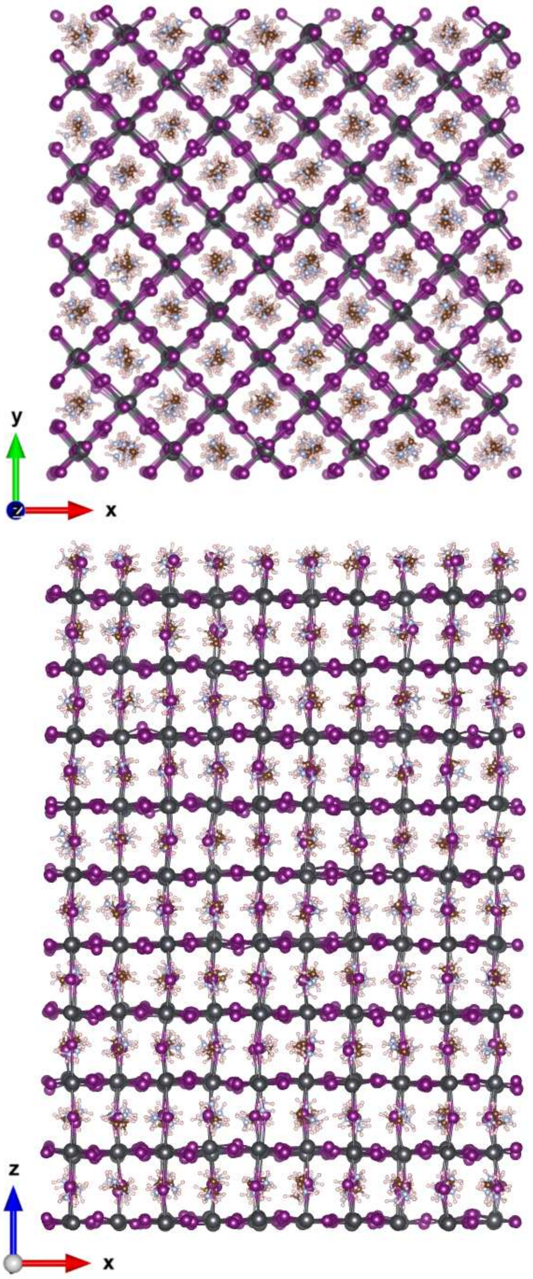

Figure S1. Ball-and-stick view for $5 \times 5 \times 5$ supercells in tetragonal phase of $MAPbI_3$.

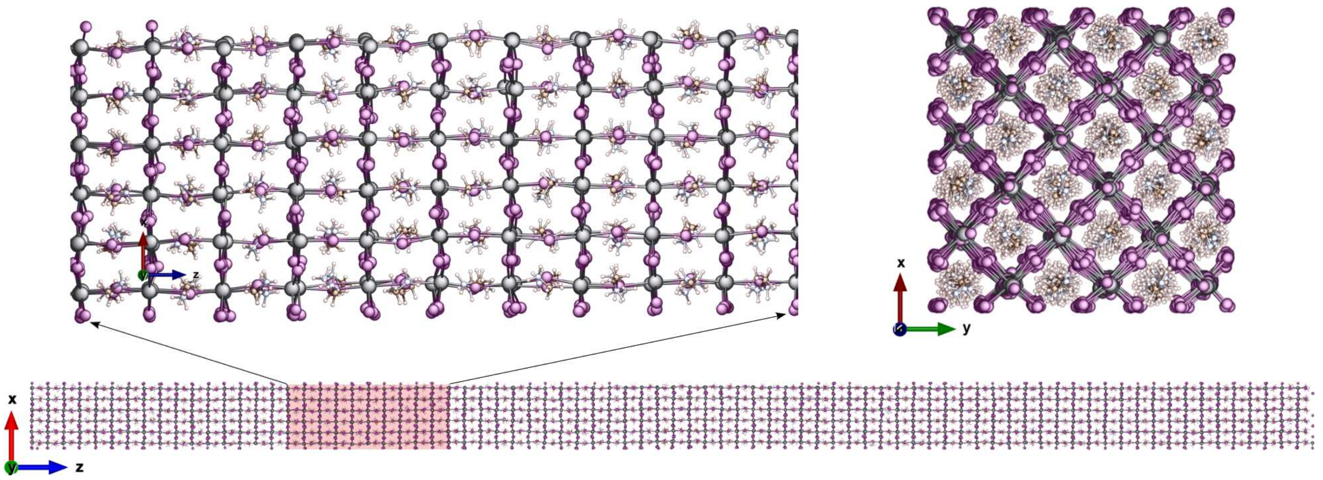

Figure S2. Ball-and-stick view for supercells with box lengths of $L_x = L_y \simeq 2.6$ nm and $L_z \simeq 51$ nm for AEMD simulations.

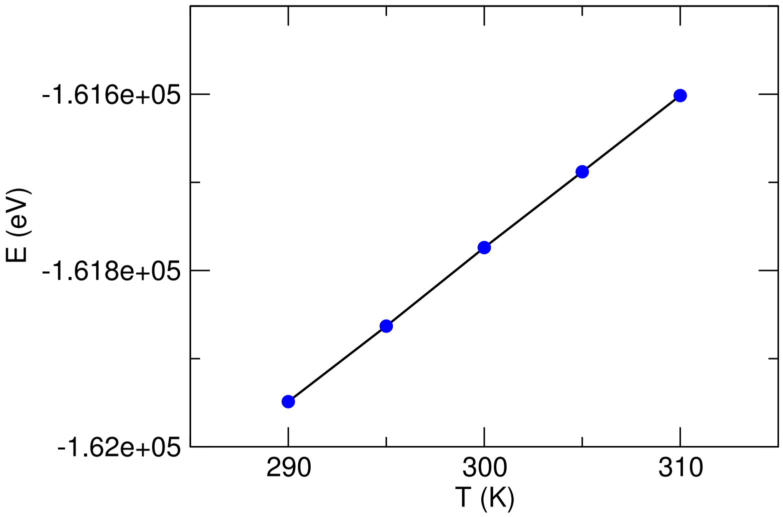

Figure S3. Total energy of the system containing 64800 atoms *vs.* temperature. Heat capacity C_V is calculated by evaluating $C_V = \frac{dE_{tot}}{dT}\Big|_V$ through calculation of the total energies at different temperatures and linear fitting.

References

[1] C. Melis, R. Dettori, S. Vandermeulen, and L. Colombo, Calculating thermal conductivity in a transient conduction regime: Theory and implementation, Eur. Phys. J. B **87**, 96 (2014).

[2] J. Feng, Mechanical properties of hybrid organic-inorganic $CH_3NH_3PbX_3$ (B = Sn, Pb; X = Br, I) perovskites for solar cell absorbers, APL Mater. **2**, 081801 (2014).

[3] Y. Rakita, S. R. Cohen, N. K. Kedem, G. Hodes, and D. Cahen, Mechanical properties of $APbX_3$ (A = Cs or CH_3NH_3; X = I or Br) perovskite single crystals, MRS Commun. **12**, 623–629 (2015).
